# Liposome-based Freezing Medium Improves the Outcome of Mouse Prepubertal Testicular Tissue Cryopreservation

**DOI:** 10.1007/s43032-024-01688-4

**Published:** 2024-09-19

**Authors:** Reyon Dcunha, Sadhana P. Mutalik, Reethu Ann Reji, Srinivas Mutalik, Sneha Guruprasad Kalthur, Padmaraj Hegde, M. S. Murari, Shamprasad Varija Raghu, Shreetama Banerjee, Anujith Kumar, Satish Kumar Adiga, Yulian Zhao, Nagarajan Kannan, Guruprasad Kalthur

**Affiliations:** 1 Division of Reproductive Genetics, Department of Reproductive Science, Kasturba Medical College, Manipal, Manipal Academy of Higher Education, Manipal, 576104 Karnataka India; 2 Department of Pharmaceutics, Manipal College of Pharmaceutical Sciences, Manipal Academy of Higher Education, Manipal, 576104 Karnataka India; 3 Centre of Excellence in Clinical Embryology, Department of Reproductive Science, Kasturba Medical College, Manipal, Manipal Academy of Higher Education, Manipal, 576104 Karnataka India; 4 Department of Anatomy, Kasturba Medical College, Manipal, Manipal Academy of Higher Education, Manipal, 576104 Karnataka India; 5 Department of Urology, Kasturba Medical College, Manipal, Manipal Academy of Higher Education, Manipal, 576104 Karnataka India; 6 DST PURSE Program, Mangalore University, Mangalagangotri, Mangalore, 574199 Karanatka India; 7 Division of Neuroscience, Yenepoya Research Centre (YRC), Yenepoya (Deemed to be University), Mangalore, 575018 Karnataka India; 8 Manipal Institute of Regenerative Medicine, Bangalore, Manipal Academy of Higher Education, Manipal, 560064 Karnataka India; 9 Division of Reproductive Endocrinology and Infertility, Department of Obstetrics and Gynecology and Department of Laboratory Medicine and Pathology, Mayo Clinic, Rochester, Minnesota 55902 USA; 10 Department of Laboratory Medicine and Pathology, Mayo Clinic, Rochester, MN 55902 USA; 11 Center for Regenerative Biotherapeutics, Mayo Clinic, Rochester, MN 55905 USA; 12 Mayo Clinic Comprehensive Cancer Center, Mayo Clinic, Rochester, MN 55905 USA; 13 Division of Reproductive Biology, Department of Reproductive Science, Kasturba Medical College, Manipal, Manipal Academy of Higher Education, Manipal, 576104 Karnataka India

**Keywords:** Oncofertility, Oxidative stress, Cryosurvival, Apoptosis, Vimentin

## Abstract

Cryopreservation of testicular tissue holds an important role in the field of fertility preservation, particularly for prepubertal boys diagnosed with cancer. However, prepubertal testicular tissue cryopreservation is still considered to be in the experimental stage necessitating the refinement of cryopreservation protocol. Considering the fact that loss of membrane lipids is the primary cause of freeze–thaw-induced loss of testicular cell functions, in this study, we explored the beneficial properties of exogenous supplementation of membrane lipids in the form of liposomes in enhancing the cryosurvival of prepubertal testicular tissue. The freezing medium supplemented with liposomes (prepared from soy lecithin, phosphatidylethanolamine, phosphatidylserine, and cholesterol) was used for the experiments. Prepubertal testicular tissues from Swiss albino mice were cryopreserved in a liposome-containing freezing medium (LFM) composed of 0.25 mg/mL liposomes, 5% DMSO, and 30% FCS in the DMEM/F12 medium using a slow freezing protocol. The tissues were thawed and assessed for various testicular cell functions. Freezing in LFM mitigated the loss of viability, decreased malondialdehyde level (*p* < 0.05), and reduced apoptosis (*p* < 0.05) in the testicular cells compared to the testicular tissue cryopreserved in the control freezing medium (CFM). Further, DMSO (5%) appears to be the ideal penetrating cryoprotectant for prepubertal testicular tissue cryopreservation with liposome-based freezing medium. Similar enhancement in cryosurvival of cells was observed in adult human testicular tissue frozen with LFM. These findings highlight the translational value of liposome-based freezing medium in the cryopreservation of testicular tissue of prepubertal boys undergoing chemotherapy.

**Supplementary Information:**

The online version contains supplementary material available at 10.1007/s43032-024-01688-4.

## Introduction

Testicular tissue cryopreservation has become integral to prepubertal cancer management in recent years. Prepubertal patients diagnosed with cancer and, undergoing chemotherapy and/or radiotherapy are the most likely candidates for testicular tissue cryopreservation [1]. Patients with life-threatening non-malignant diseases, such as thalassemia, drepanocytosis, granulomatous, and idiopathic medulla aplasia, undergo whole-body irradiation as a conditioning therapy before undergoing bone marrow transplantation and therefore carry a significant risk of infertility. In addition, in genetic and congenital conditions like Klinefelter syndrome and gender dysphoria, testicular tissue cryopreservation is offered at a young age, before initiating hormone therapy as a precautionary measure [1, 2].

Testicular tissue cryopreservation involves excising a small piece of the testicular tissue and freezing it for future use. If the patient reaches adulthood and wishes to have a biological child from his spermatozoa, the cryopreserved tissue can be thawed to derive the spermatozoa. However, protocols to derive spermatozoa from prepubertal testicular tissue through in vitro spermatogenesis or xenotransplantation/ auto transplantation techniques are still in their early stages of development [3].

One major reason for the poor prepubertal testicular tissue cryopreservation outcome is the compromised functional properties of the testicular cells. Several approaches have been explored previously to optimize the slow freezing and vitrification protocols for testicular tissue cryopreservation. However, it is well established that irrespective of the type of freezing protocol employed, the freeze–thaw process causes oxidative stress [4], structural damage to proteins [5], loss [6–8] and rearrangements [9, 10] of membrane lipids, and DNA damage [11], collectively impairing cellular function. Therefore, to understand the outcome of freeze–thaw process on testicular tissue, the expression pattern of genes involved in the regulation of apoptosis (BAX, BCL2, P53, Cytochrome c, Caspase-3), oxidative stress markers [Superoxide dismutase 1 (SOD1), catalase (CAT), glutathione peroxidase (GPX), glutathione (GSH), malondialdehyde (MDA), protein carbonylation (PCO) and DNA damage, are commonly studied [12–14]. Mitigation of oxidative stress and membrane damage has been considered as an ideal strategy to overcome the freeze–thaw-induced loss of cellular function [15–17]. Earlier studies have demonstrated that the improvement in the freeze–thaw process of testicular tissue by the use of additives or antioxidant molecules in the freezing medium was associated with a decrease in MDA level, an increase in BCl2, and reduced Caspase-3 expression [18, 19].

Intuitively the presence of membrane lipids and free radical scavengers in the freezing media is expected to enhance the cryopreservation outcome. To prevent the loss of functional competence of the cells during the freeze–thaw process, exogenous supplementation of membrane lipids in the form of liposomes has been successfully attempted earlier [20, 21]. Experimental evidences suggests that characteristic features of liposomes such as, high lipid content, stability in low temperature and lamellarity, and their ability to interact with cell membrane helps in improving the cell survival in biological specimens during freeze–thaw process [22, 23]. In an earlier study, we demonstrated that cryopreservation of human semen samples in freezing media supplemented with liposomes rich in membrane phospholipids prevents the loss of motility and viability in spermatozoa [20]. Similarly, micelles composed of glycerophospholipids are reported to mitigate oxidative stress generated during the freeze–thaw process [24]. Based on these findings from the literature, the present study aimed to explore the beneficial effect of freezing medium composed of liposomes prepared from membrane lipids in improving the outcome of prepubertal testicular tissue cryopreservation.

## Materials and Methods

### Testicular Tissue from Mice and Human

Inbred prepubertal Swiss albino male mice (two weeks) were procured from the Central Animal Research Facility of the Manipal Academy of Higher Education, Manipal, India to obtain testicular tissues. The current investigation was approved in advance by the Kasturba Medical College, Manipal Institutional Animal Ethical Committee (IAEC/KMC/18/2015; IAEC/KMC,134/2019). The procedures used in this investigation were compliant with the national and institutional criteria for the supervision of experiments on animals (CPCSEA) and with the ARRIVE guidelines.

Adult human testicular tissues collected from prostate cancer patients (N = 6, 69.50 ± 2.26 years) undergoing orchiectomy at the Department of Urology, Kasturba Medical College, Manipal, India, were used for this study. The subjects who agreed to donate their testicular tissue for the study signed a written consent form. Prior approval from the Institutional Ethics Committee (IEC 874/2020) of Kasturba Medical College and Kasturba Hospital, Manipal, Manipal Academy of Higher Education, Manipal, was taken for the study.

### Preparation of Liposomes

Two types of liposomes, simple and complex were prepared by thin-film hydration method. The mixture of constituents used for the preparation of simple liposomes and liposomes encapsulating sodium selenite (214485, Sigma-Aldrich, USA) and vitamin C (A4544, Sigma-Aldrich, USA; complex liposomes) is mentioned in Table 1. The concentration of these lipids is based on an earlier preliminary study (data not shown). The constituents were dissolved in 10 mL chloroform, in a round bottom flask. The solvent was evaporated using a rotary flash evaporator (Buchhi Rotavapor) at 45ºC to form a thin film. Residual solvents were removed by placing the flask in a desiccator under vacuum overnight. Next, the lipidic thin film was hydrated using 10 mL phosphate buffer at 55ºC. Further, the liposomal suspension in the round bottom flask was subjected to bath sonication till the film was completely peeled off. Thus, the obtained liposomal suspension was subjected to probe sonication to obtain colloidal suspension with a uniform size distribution of particles. Parameters of probe sonication such as pulse (5 s on-2 s off) and sonication time (6 min) were optimized to obtain an optimum size of around 200 nm. To prepare complex liposomes, sodium selenite, and vitamin C at their optimum concentration were dissolved in 10 mL phosphate buffer (pH 7.4), which was used as a hydration medium, and the rest of the procedure was the same as explained above. The liposomes containing fluorescein 5(6)-isothiocyanate (FITC; 46950, Sigma-Aldrich, USA) were also prepared similarly.

**Table 1:** Composition of the simple and complex liposomes

Components	Quantity (mg/ mL)
Simple liposomes	
Soy lecithin	2.5
Cholesterol	1.0
Phosphatidylethanolamine	1.0
Phosphatidylserine	0.25
Complex liposomes	
Soy lecithin	2.5
Cholesterol	1.0
Phosphatidylethanolamine	1.0
Phosphatidylserine	0.25
Vitamin C	0.6
Sodium selenite	0.004

### Cryopreservation and Thawing

**Fig. 1:**
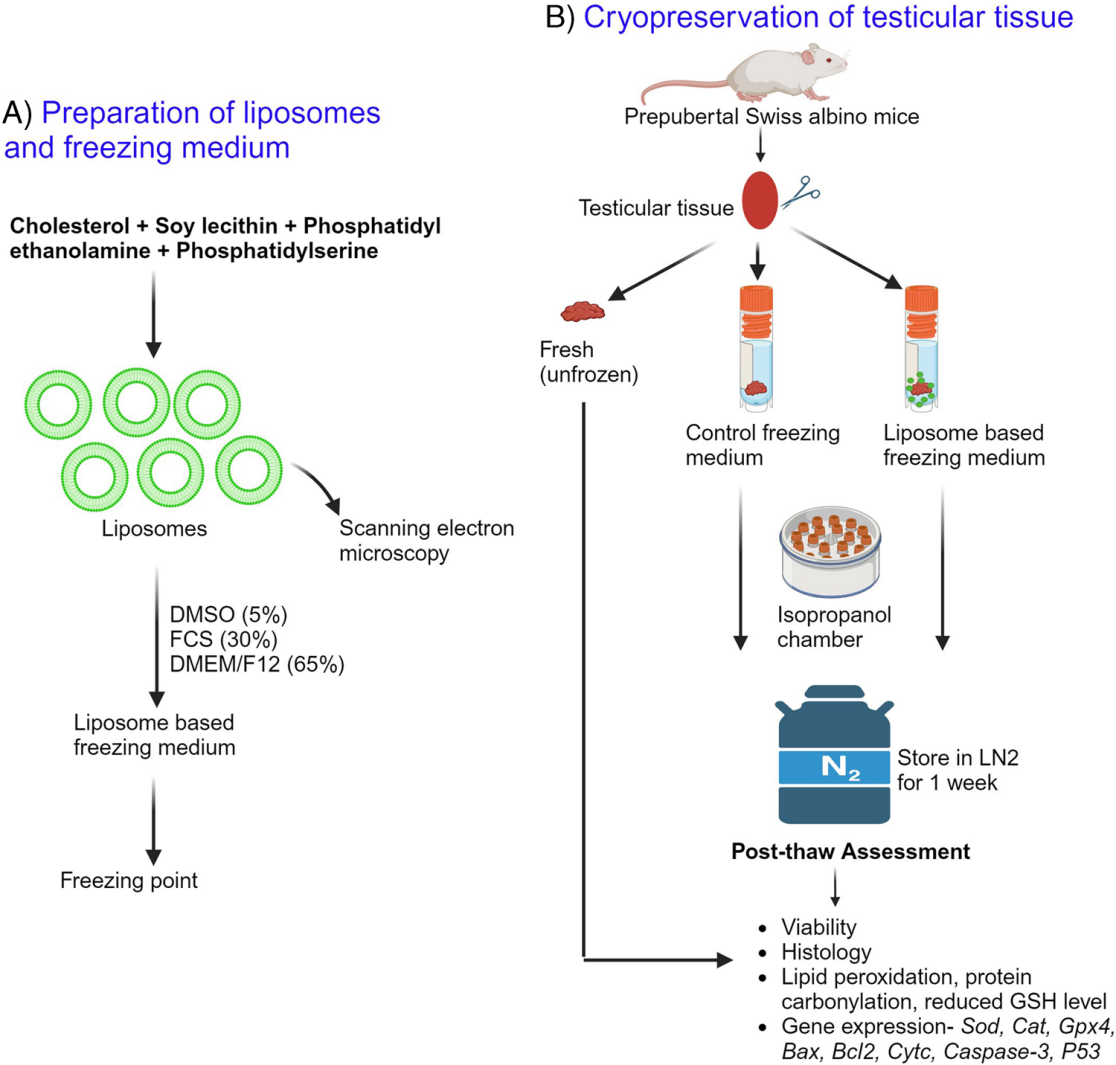
**A)** Schematic representation showing the preparation of liposome-based freezing medium for cryopreservation of mouse prepubertal testicular tissue. **B)** Experimental outline to understand the effect of liposome-based freezing medium on prepubertal testicular tissue cryopreservation. The images were created with Biorender.com

Testicular tissue cryopreservation was performed by slow freezing protocol, as previously described [25], with few modifications. Briefly, the testicular tissue was collected in DMEM/F12 (11320033, Gibco, USA) medium on ice, after euthanizing the mice by cervical dislocation. Immediately after collection, the testis was decapsulated and cut into 3 mm^2^ pieces. The testicular tissue was then transferred to a cryovial (P60116, Abdos, USA) containing 500 μL of either control freezing medium (CFM) composed of 5% dimethyl sulfoxide (DMSO, D4540, Sigma, USA) and 30% fetal bovine serum (FBS; CCS-500-SA-U, Genetix Biotech, Cell clone™, India) in DMEM/F12 medium or the liposome freezing medium (LFM) composed of 2.5 mg/mL soy lecithin (P5638, Sigma-Aldrich, USA), 1.0 mg/mL phosphatidylethanolamine (P1223, Sigma-Aldrich, USA), 0.25 mg/mL phosphatidylserine (P7769, Sigma-Aldrich, USA), 1.0 mg/mL cholesterol (10367201001730, Merck, India), 5% DMSO and 30% FBS in DMEM/F12 medium (Fig. 1A; Patent applied, 202341036047). The cryovials were then placed in an isopropanol chamber (Mr. Frosty™ Freezing Container, 5100–0001, Thermofisher Scientific, USA) at -80ºC for 24 h and then stored in liquid nitrogen (LN_2_) for a minimum of one week (Fig. 1B).

Thawing of testicular tissue was performed according to the protocol described by Milazzo et al. [26], with minor modifications. Briefly, the samples were thawed rapidly by placing the cryovials in a water bath maintained at 37ºC for 2 min. Tissues were then placed sequentially in thawing solution 1 (TS1; 2.5% DMSO, 0.05 M sucrose and 10% FBS in DMEM/F12 medium) followed by thawing solution 2 (TS2; 1% DMSO, 0.05 M sucrose and 10% FBS in DMEM/F12 medium), thawing solution 3 (TS3; 0.05 M sucrose in DMEM/F12 medium) and finally in thawing solution 4 (TS4; DMEM/F12 Medium) for 5 min each at room temperature. The tissues were kept on ice until further handling.

### Characterization of Liposomes

Simple liposomes and complex liposomes were diluted ten times with Milli-Q® (ZR0Q008WW, Merck, USA) water and characterized for size and zeta potential using Zetasizer (Nano ZS, Malvern Instruments, UK).

#### Scanning Electron Microscopy (SEM) Analysis

The lyophilized liposome samples were finely powdered in agate mortars for 2 min. Small quantities of samples were ultra-sonicated for 10 min to get a uniform distribution of particles. The samples were dried in a vacuum desiccator for 10 min. The surface morphology of the samples was studied in 03–81 models, Carl Zeiss Field Emission Scanning Electron microscope (FESEM) at the DST-PURSE facility, Mangalore University.

#### Construction of Calibration Curve for Vitamin C by UV–Vis Spectroscopy

To find out the wavelength of maximum absorbance (λ_max_) of vitamin C, a spectral scan ranging from 200 to 800 nm was performed using a UV/Vis spectrophotometer (UV-1601PC, Shimadzu, Japan). A stock solution of vitamin C was prepared in distilled water to obtain a concentration of 1 mg/mL. A standard solution of 10 μg/ mL was obtained with appropriate dilution with phosphate buffer (pH 7.4) and was used for the spectral scan. A calibration curve was constructed with six standard points of concentration 2, 5, 8, 10, 12, and 14 μg/ mL constructed with phosphate buffer (pH 7.4) as blank. The regression coefficient was obtained, and the slope was determined by the linear equation.

#### Entrapment Efficiency

To estimate the entrapment efficiency, the liposomal suspension was centrifuged at 2200 × g at 4ºC for 1 h. The supernatant was discarded and the pellet was re-dispersed in 1 mL of phosphate-buffered saline (PBS, pH 7.4). Next, to 100 μL of the suspension, 10% Triton-X was added and subjected to water bath sonication for 2 min and the volume was made up to 1 mL with PBS. The mixture was centrifuged at 100 × g for 10 min and the supernatant was appropriately diluted with PBS. The absorbance of the sample was analyzed using a UV–Vis spectrophotometer. The entrapment efficiency was calculated using a formula:

$$\%\;Entrapment\;efficiency=\frac{Entrapped\;drug}{Total\;drug}\times\;100$$

### Enzymatic Digestion to Collect Testicular Cells

Testicular cells were isolated using the methods described earlier [27], with minor modifications. Briefly, the de-capsulated testes were suspended in DMEM containing 1 mg/mL trypsin (49,041, Gibco, USA) and collagenase type IV (17,104–019, Gibco, USA) and were incubated at 37ºC for 30 min with intermittent shaking. DMEM containing 10% FCS (CCS-500-SA-U, Genetex, India) was used to neutralize trypsin action and the cells were filtered using 70 μm cell strainer (22–363-548, Fisher Scientific, USA) followed by filtration using a 40 μm cell strainer (22–363-547, Fisher Scientific, USA) after which the cells were centrifuged at 100 × g for 10 min. The cell pellet was re-suspended in a minimum amount of PBS and used for further assessment. The number of viable cells was determined by the trypan blue dye exclusion method immediately after cell isolation from the tissue. A hemocytometer was used to manually count the percentage of viable cells after being stained with 0.4% trypan blue.

### Histology and Immunohistochemistry

To assess the histological changes after the freeze–thaw process, testicular fragments were first fixed for 48 h in Bouin’s fixative, followed by storage in 70% ethanol. The tissues were then successively dehydrated using graded alcohol before embedding in paraffin. 5 μm thick sections were prepared, stained with hematoxylin and eosin, and observed under a light microscope at 400 × magnification.

The procedure for immunohistochemical staining was followed as described earlier [28]. In brief, the slides were deparaffinized in a dry bath at 60ºC. Next, the slides were immersed in xylene for 15 min, followed by serially dehydrating the slides for 2 min in ethanol concentrations of 100, 95, and 70%. After that, the slides were submerged in 0.01 M sodium citrate buffer (pH 6.0) and heated to a boiling point for 20 min for antigen retrieval. Blocking was performed with 1% bovine serum albumin (BSA) for 1 h at 37ºC after which the slides were incubated with an anti-vimentin primary antibody (1:200; 5741, Cell Signaling Technology, USA) overnight at 4ºC. After washing in PBS thrice, the sections were incubated with goat anti-rabbit Alexa fluor™ 488 secondary antibody for 1 h at 37ºC. Excess and non-specific binding was removed by thoroughly washing the slides with PBS. The sections were then mounted using Fluoroshield™ with DAPI (F5932, Sigma-Aldrich, USA) and imaged using QCapture Pro 7 software, USA, equipped with a fluorescence microscope.

### Extraction and Estimation of Protein in Testicular Tissue

Testicular tissue homogenate was prepared in PBS using hand homogenizer. After estimating the protein level by Bradford’s method [29], the oxidative stress generated during the freeze–thaw process was evaluated by measuring the MDA, GSH, and PCO content in the testicular homogenate as described below.

### Estimation of Malondialdehyde (MDA) in Testicular Tissue

MDA estimation was performed as described earlier [30].Two parts of testicular homogenate were mixed with one part of precipitating reagent containing equal volumes of trichloroacetic acid [TCA, (15% in 0.25 N HCl) and 2-thiobarbituric acid (TBA, 0.37% in milli-Q water)]. The mixture was kept in a water bath maintained at 90ºC for 15 min followed by cooling on ice. The precipitate formed was removed by centrifugation at 500 × g for 15 min. The absorbance of the red-colored complex was measured at 550 nm against the reagent blank. MDA concentration was expressed in Mol/ mg protein.

### Estimation of Reduced Glutathione (GSH)

GSH estimation in the testicular homogenate was performed using the method described earlier [31], with minor modifications. Briefly, 5% of TCA was added to the testicular homogenate to precipitate the proteins and then centrifuged at 400 × g for 5 min at 4ºC to obtain the supernatant. The collected supernatant was mixed thoroughly with 0.1 M potassium phosphate buffer and 2 mM DTNB [5,5-dithiobis (2-nitro-benzoic acid), D8130, Sigma-Aldrich, USA] containing 50 mM sodium acetate and the optical density (OD) of the colored complex formed were taken at 405 nm using a Biophotometer plus (Eppendorf, Germany). The GSH level was calculated by plotting the standard graph against the OD values of GSH ranging from 1.5 to 20 μg/mL. The GSH concentration was expressed as μM/mg of the protein.

### Estimation of Protein Carbonyl Content (PCO)

PCO was estimated as described earlier [30] using the 2,4-dinitrophenyl hydrazine (DNPH) method. Briefly, 10 mM DNPH solution was added to the homogenate samples. Blank was prepared by adding 2 N HCl without DNPH to the homogenate. After vortexing, the samples were incubated in the dark at room temperature for 1 h, followed by incubation with 20% TCA solution for 15 min on ice. The samples were centrifuged at 10,000 × g for 5 min at 4ºC to obtain the protein pellet. The obtained protein pellet was washed with 20% TCA followed by 1:1 (v/v) ethanol: ethyl acetate to remove traces of DNPH and dried for 5 min to remove the solvents. The dried pellet was then resuspended in 6 M guanidine hydrochloride (38487-K01, S.D. Fine-Chem, India) prepared in 50 mM phosphate buffer at pH 2.3 and incubated at 37ºC for 15–30 min with vortex mixing to dissolve the proteins completely. The carbonyl content in the sample was determined at ~ 366 nm using a molar extinction coefficient of 22,000 M^−1^ cm^−1^ against blank. The results were expressed as nM of carbonyl /mg protein using the formula mentioned below.

$$Carbonyl\;content\;of\;sample\;\left(nM/mgof\;protein\right)=\frac{\left[\left(Absorbance\;at\;366\;nm\;\times \text{22,000} {M}^{-1} {cm}^{-1}\right)\times {10}^{6}\right]}{Protein\;concentration\;in\;mg/mL}$$

#### Immunofluorescence

The cell suspension was fixed with 4% paraformaldehyde (PFA) overnight at 4ºC, washed with PBS, and then smeared on a clean glass slide. The cells were subsequently permeabilized for 10 min with 0.3% Triton X-100 in PBS, rinsed in PBS, and then blocked for 30 min with PBS containing 1% BSA and 0.1% Tween-20. Next, primary antibodies: Anti-phosphohistone H2AX (1:250; 9718S, Cell Signaling Technology, USA), Annexin V (1: 200; 8555S, Cell Signaling Technology, USA) in PBST (PBS containing 0.1% Tween-20) were added and incubated overnight at 4ºC. Then, the cells were mounted using Fluoroshield™ with DAPI after being treated with goat anti-rabbit Alexa fluor™ 488 secondary antibody (ab150077, Abcam, UK) for 1 h at 37ºC. Excess and non-specific binding was removed by thoroughly washing the slides with PBS. Cells were visualized under a fluorescence microscope and the images were captured using QCapture Pro 7 software, USA. A minimum of 500 cells were counted under 400 × magnification, and the data was reported as a percentage of the cells with positive signals for the protein of interest.

### Gene Expression Analysis

Gene transcription analysis for Tumor protein P53 (*p53*), Apoptosis regulator BAX (*Bax*), B-cell lymphoma 2 (*Bcl-2*), Cytochrome c (*Cyt c*), Caspase-3, Superoxide dismutase (*Sod1*), Glutathione peroxidase 4 (*Gpx4*) and Catalase (*Cat*) was performed using a real-time PCR as described earlier [32]. Briefly, testicular tissue was dissolved in TRI reagent® (T9424, Sigma-Aldrich, USA). After the tissue dissolved completely, chloroform was added, vigorously mixed for 5 min and the contents were centrifuged at 12,000 × g for 15 min at 4ºC. The top clear aqueous layer was transferred to a sterile diethylpyrocarbonate (DEPC) treated microfuge tube and isopropanol was added to precipitate the RNA. Next, the contents were mixed thoroughly and allowed to stand at room temperature for 5 min following which the mixture was centrifuged at 12,000 × g for 15 min at 4ºC. The obtained pellet was washed with 75% ethanol and centrifuged at 7500 × g for 5 min at 4ºC. After the pellet was semi-dried, Rnase-free water was added and allowed to stand for 5 min at room temperature to ensure complete dissolution of RNA. The RNA was stored at -80ºC until further analysis.

The RNA quantification was performed using the BioPhotometer Plus (Eppendorf, Germany). High-capacity cDNA Reverse Transcription Kit (4368814, Applied Biosystems, USA) was used to synthesize cDNA according to the manufacturer's instructions. The relative gene expression was carried out using the StepOne real-time PCR system (Applied Biosystems, USA) using TaqMan® assay probes and SYBR Green chemistry (RR390A, TaKaRa, Shiga, Japan). The amplification was carried out for 20 s incubation at 95ºC, amplification at 95ºC for 1 s, and annealing at 60ºC for 20 s for 40 cycles. The fluorescence emitted at each cycle was collected for an entire period of 30 s during the extension step of each cycle. Mean Ct Values were generated and the cDNA concentrations in the sample were computed and normalized to Glyceraldehyde 3-phosphate dehydrogenase (*Gapdh)*. The relative expression levels in terms of fold change were calculated by the 2^−ΔΔCt^ method. All experiments were performed in triplicates. The list of primers used in the study is provided in Table S1 (Supplementary information).

### Western Blot Analysis

Tissues were homogenized using a probe sonicator in RIPA lysis buffer (R0278, Sigma-Aldrich, USA), and were centrifuged at 12,000 × g for 15 min at 4ºC. Protease inhibitor cocktail (P2714, Sigma-Aldrich, USA) was added to the buffer prior to lysis. The supernatant was collected and stored at -80ºC until further use. Proteins were resolved on a 12% SDS-PAGE and transferred to an activated PVDF membrane for 1.5 h at 100 mA using a semi-dry transfer apparatus. The blots were blocked using 3% skimmed milk and later incubated with β-Actin Mouse mAb (AC004, ABclonal, USA), SOD1(CSB-PA02864A0Rb, CUSABIO, USA), Cytochrome c Oxidase βA1 (CSB-PA02315A0Rb, CUSABIO, USA) and Caspase-3 (CSB-PA05689A0Rb, CUSABIO, USA) antibodies at a dilution of 1:1000 overnight at 4ºC on a rocking platform. Three washes were performed using 1X TBST (pH 7.4) followed by incubation for 2 h with HRP-tagged anti-rabbit secondary antibody (111,035,003, Jackson ImmunoResearch, USA) or HRP-tagged anti-mouse secondary antibody (62–6520, Invitrogen, USA) at a final concentration of 1:2000 in skimmed milk at room temperature. After three washes with 1X TBST, the signal was developed using WesternBright ECL substrate (K120445- D20, Advansta, USA) and captured in Chemidoc XRS + .

### Freezing Point Determination

Ice cubes were crushed into small pieces and mixed with sodium chloride to prepare the ice-salt mixture. An alcohol thermometer placed in the ice-salt mixture was used to monitor the temperature below 0ºC. To estimate the freezing point of the cryopreservation medium, 2 mL of either CFM or LFM was taken in a test tube and placed in a container with an ice-salt mixture. Precautions were taken to ensure that the medium in the test tubes was below the level of the ice-salt mixture. Milli-Q® water was used as a positive control. The freezing media and MilliQ water froze when placed in the ice-salt mixture. As the ice-salt mixture started melting, the temperature of the mixture increased towards 0ºC. The frozen state of the samples was continuously monitored as the temperature increased using capillary tubes. When the samples turned from solid ice to liquid crystal form, the temperature (freezing point) was recorded.

### Statistics

All the numerical data are expressed as the mean and standard error of the mean (Mean ± SEM). GraphPad Prism 8.0 (GraphPad Software Inc., USA) was used to analyze the data. One-way analysis of variance (ANOVA) was performed to determine the statistical comparison among the groups, followed by Tukey’s multiple comparison test. For grouped analysis, two-way ANOVA was performed followed by Tukey’s multiple comparison test. A p-value ≤ 0.05 indicated statistical significance.

## Results

### Characterization of Liposomes

**Fig. 2:**
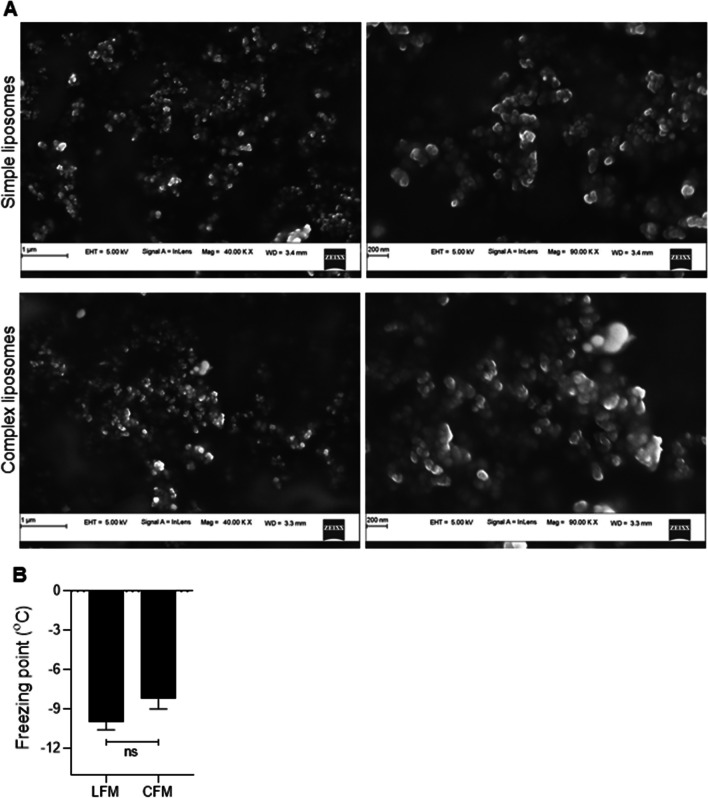
**A)** Surface electron microscopic images showing equal size distribution in the simple and complex liposomes prepared from membrane lipids and antioxidants. **B)** Lower freezing point of liposome-based cryopreservation medium (LFM) compared to the DMSO-based freezing medium (CFM)

The simple as well as complex liposomes exhibited spherical-shaped vesicles, with almost equal size distribution (Fig. 2A), which was further confirmed using the ZetaSizer. The size of the simple liposomes was found to be 205.00 ± 2.34 nm and the complex liposomes were 181.30 ± 1.96 nm in size, with a polydispersity index of < 0.5 and < 0.2, respectively, indicating homogeneity of liposomes. The zeta potential was found to be -59.90 ± 0.76 mV for simple liposomes and -52.95 ± 1.08 mV for complex liposomes indicating a stable colloidal property of the prepared liposomal suspension (Table 2). Further, the preparation of liposomes encapsulated with vitamin C and selenium did not affect the size of the vesicles considerably. The absorbance maximum (λmax) of vitamin C was observed at 265 nm. The calibration curves (N = 3) plotted with the concentration *vs*. absorption for a range between 2–14 μg/mL yielded an r^2^ value of 0.998 and a linear equation (y = 0.0691x—0.0008) was obtained. The entrapment efficiency of the liposomal suspension as measured by UV absorbance was found to be 12.23% with a vitamin C concentration of 1.467 mg/mL of the liposomal suspension (Table 2).

**Table 2:** Characterization of simple and complex liposomes

Parameter	Value	
	Simple Liposomes	Complex Liposomes
Size (nm)	205 ± 2.34	181.3 ± 1.96
Polydispersity index	< 0.5	< 0.2
Zeta potential (mV)	-59.9 ± 0.76	-52.95 ± 1.08
Encapsulation efficiency (%)	Not applicable	12.23 ± 2.34

### Freezing Point of Liposome-based Medium

Preliminary screening with different concentrations of simple liposome (0.05 to 5 mg/mL) in the cryopreservation medium revealed a maximum cryoprotective effect at 0.25 mg/mL concentration on the testicular cells with respect to cell viability and DNA integrity (Fig S1A-D, Supplementary information). Complex liposomes did not show any additional benefits over simple liposomes in enhancing the cryosurvival of testicular cells. Therefore, 0.25 mg/mL of simple liposomes (LFM) was used for further experiments. Since the freezing point of the cryopreservation medium can change with the composition of the freezing medium we wanted to test whether the presence of liposomes in the freezing medium alters the freezing point. Compared to the CFM, the freezing point of LFM was observed to be non-significantly lower (Fig. 2B).

### Effect of Liposome-based Medium on the Outcome of Prepubertal Mice Testicular Tissue Cryopreservation

**Fig. 3:**
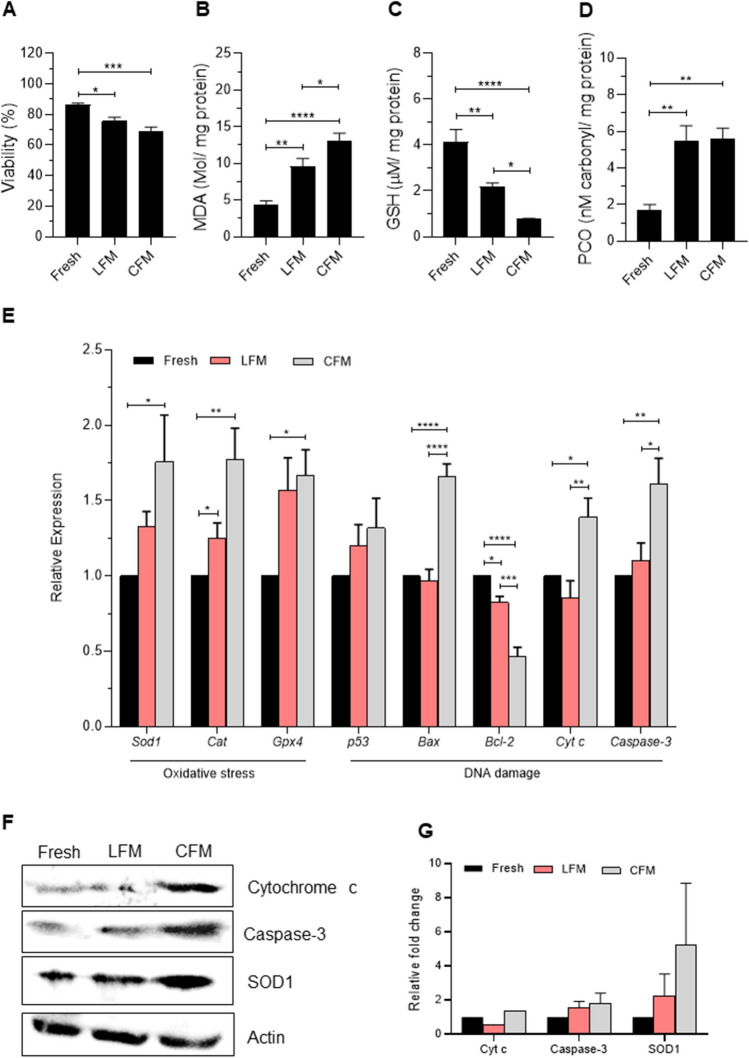
Outcome of prepubertal testicular tissue cryopreservation using a liposome-based freezing medium. **A)** Effect of LFM on cell viability assessed by trypan blue dye exclusion method in prepubertal testicular tissue subjected to cryopreservation by slow freezing method (*N* = 26). *p < 0.05, ***p < 0.001 v/s Fresh. Assessment of **B)** Malondialdehyde, **C)** Reduced glutathione, **D)** Protein carbonyl content in prepubertal mice testicular tissue cryopreserved with CFM and LFM (*N* = 6). **p < 0.01, ****p < 0.0001 v/s Fresh, *p < 0.05 v/s CFM. **E)** Gene expression analysis for *Sod1*, *Cat*, *Gpx4, p53*, *Bcl-2*, *Bax*, *Cytc,* and *Caspase-3* in the prepubertal testicular tissues cryopreserved with CFM and LFM analyzed by qRT-PCR (*N* = 6). *p < 0.05, **p < 0.01, ****p < 0.0001 v/s Fresh, *p < 0.05, **p < 0.01, ***p < 0.001, ****p < 0.0001 vs/ CFM. **F)** Protein expression of SOD1, Caspase-3, and Cytochrome c oxidase in different conditions and, **G)** Densitometric quantification of proteins relative to β-Actin from the blots represented in F (*N* = 3). The data is represented as Mean ± SEM

Cryopreservation of prepubertal testicular tissue in CFM resulted in a significant decrease in viability of testicular cells (*p* < 0.001) compared to fresh tissue (Fig. 3A). However, cryopreservation of prepubertal testicular tissue in LFM prevented the loss of cell viability, even though the difference was statistically not significant. During the freeze–thaw process, a significant increase (*p* < 0.0001) in MDA, and PCO content (*p* < 0.01) and a decrease (*p* < 0.0001) in reduced GSH levels was observed in the tissue frozen with CFM compared to the fresh tissue. Comparatively, cryopreserving the testicular tissue in LFM resulted in a significant decrease in MDA level (*p* < 0.05; Fig. 3B) and a higher level of reduced GSH (*p* < 0.05; Fig. 3C). However, PCO content did not differ between tissues frozen with CFM and LFM (Fig. 3D).

Gene expression analysis for key antioxidant markers further confirmed these findings. Cryopreservation of testicular tissue in CFM resulted in a significant increase in mRNA transcripts for *Sod1* (*p* < 0.05), *Cat* (*p* < 0.01), and *Gpx4* (*p* < 0.05) compared to the fresh tissue. While in the tissues cryopreserved with LFM, the decreased expression of *Cat* (*p* < 0.05), *Sod1,* and *Gpx4* (both non-significant) was observed compared to CFM (Fig. 3E). At the protein level, a three-fold increase in SOD1 level was observed in the tissues frozen in CFM compared to the fresh tissue, which was lower in the tissue cryopreserved in the LFM (Fig. 3F-G).

**Fig. 4:**
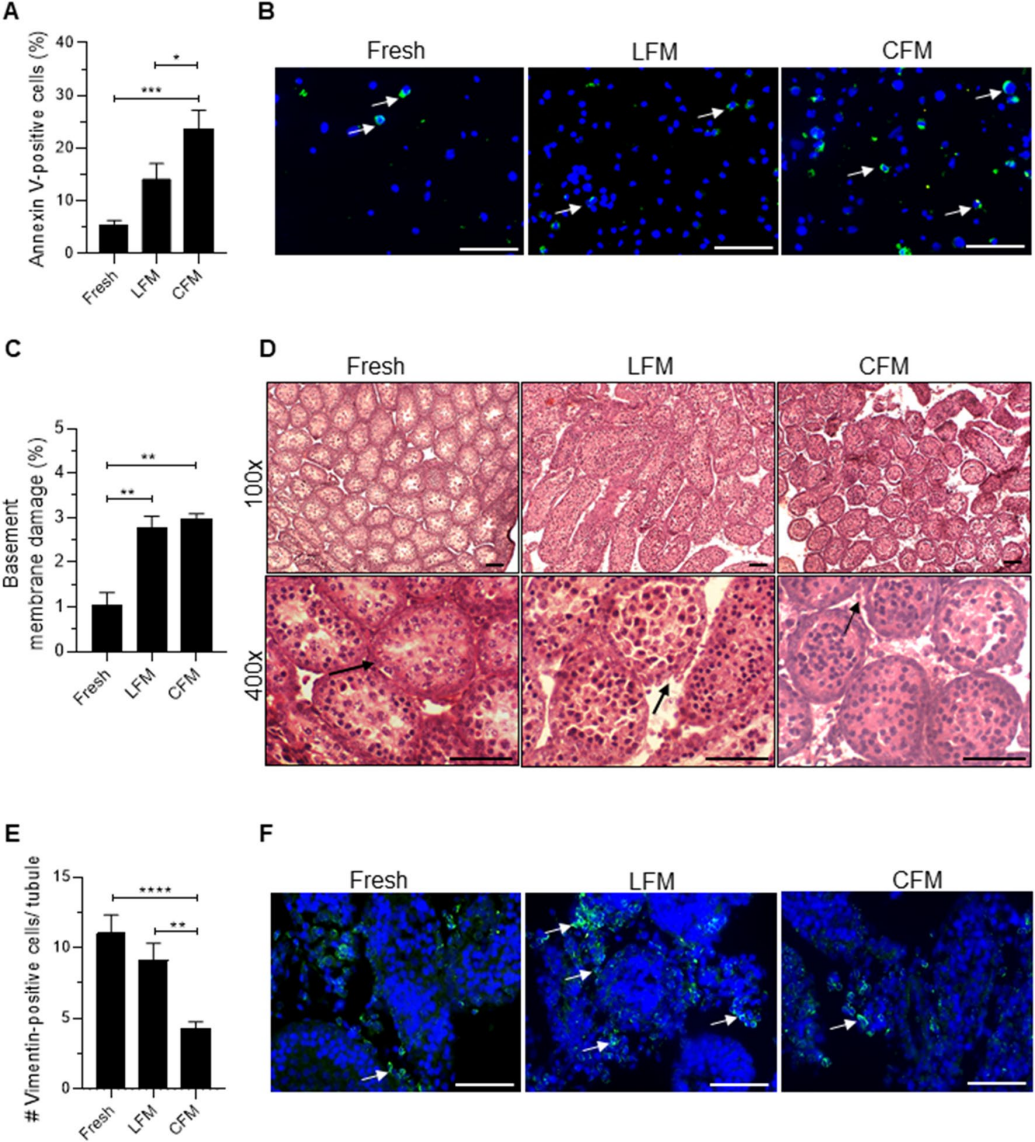
**A)** Assessment of apoptosis by Annexin V expression in prepubertal testicular tissue subjected to cryopreservation by slow freezing method (*N* = 9); ***p < 0.0001 vs/ Fresh, *p < 0.05 v/s CFM. **B)** Representative merged images for Annexin V-positive testicular cells, nucleus counterstained with DAPI, 400 × magnification; White arrow represents the Annexin V-positive cells; Scale bar represents 50 μm. **C)** Incidence of basement membrane damage in prepubertal testicular tissue subjected to cryopreservation with CFM and LFM (*N* = 3). **p < 0.01 v/s Fresh, **p < 0.01 v/s CFM. Representative images to depict basement membrane damage in prepubertal mice testicular tissue cryopreserved with CFM and LFM, **D)** at 100 and 400 × magnifications; Scale represents 50 μm; Arrow indicates basement membrane integrity. **E)** Expression of vimentin in prepubertal testicular tissue after freeze–thaw process (*N* = 3). ****p < 0.0001 v/s Fresh, **p < 0.01 v/s CFM. The data is represented as Mean ± SEM. **F)** Representative merged images for vimentin-positive testicular cells, nucleus counterstained with DAPI, 400 × magnification; Arrow represents the vimentin-positive cells; the scale bar represents 50 μm

Further, assessment of genes involved in the apoptotic pathway revealed a significant increase in the mRNA transcripts of *Bax* (*p* < 0.0001), *Cyt c* (*p* < 0.05), and *Caspase-3* (*p* < 0.01) in the tissues frozen with CFM compared to fresh tissue. In comparison, a significant decrease in mRNA transcripts of *Bax* (*p* < 0.0001), *Cyt c* (*p* < 0.01), and *Caspase-3* (*p* < 0.05) was observed in tissue cryopreserved with LFM. In addition, a significant increase in *Bcl-2* (p < 0.001) was observed in tissue cryopreserved in LFM. However, no change in the expression of *p53* was observed in the CFM and LFM (Fig. 3E). Similar expression pattern was observed for proteins involved in the apoptotic pathway. The number of Annexin V-positive cells, an early apoptotic marker, was observed to be significantly higher (*p* < 0.001) in testicular cells of tissues frozen in CFM compared to fresh tissue, which was significantly lower (*p* < 0.05) when tissues were cryopreserved with LFM (Fig. 4A, B). Similarly, Cytochrome c and Caspase-3 expression were higher in tissue cryopreserved in CFM compared to the fresh tissue. Although the expression was reduced in the LFM it was non-significant (Fig. 3F-G).

### Histological Organization of Prepubertal Testicular Tissue Cryopreserved in LFM

**Fig. 5:**
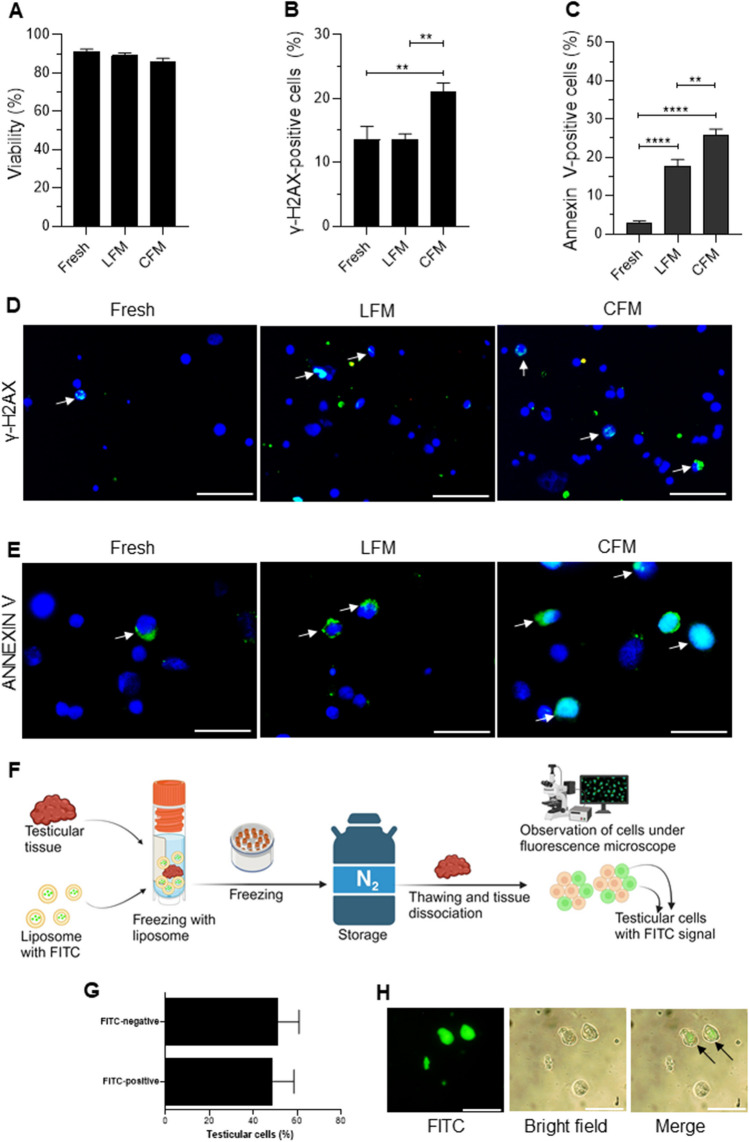
Effect of freeze–thaw process on the outcome in human adult testicular tissues cryopreserved with CFM or LFM. **A)** Viability, **B)** DNA damage assessed by γ-H2AX expression; **p < 0.01 v/s Fresh, **p < 0.01 v/s CFM. **C)** Incidence of apoptosis assessed by Annexin V expression in testicular cells; ****p < 0.0001 v/s Fresh, **p < 0.01 v/s CFM. **D)** Representative merged images for testicular cells positive for γ-H2AX, nucleus counterstained with DAPI, 400 × magnification; the arrow represents the γ-H2AX-positive cells; scale bar represents 50 μm. **E)** Representative merged images for testicular cells positive for Annexin V, nucleus counterstained with DAPI, 1000 × magnification; the arrow represents Annexin V-positive cells; scale bar represents 20 μm. The data is represented as Mean ± SEM (*N* = 6). **F)** Schematic representation of the method followed to assess the uptake of fluorescence (FITC) labeled liposomes in the freezing medium by testicular cells (created with Biorender.com). **G)** Percentage of FITC-positive testicular cells cryopreserved with freezing medium containing fluorescence (FITC) labeled liposomes. **H)** Representative images of FITC-positive and negative testicular cells cryopreserved with freezing medium containing fluorescence (FITC) labeled liposomes. Arrows indicate cells that have taken up the fluorescent dye, 400 × magnification; scale bar represents 50 μm. The data is represented as Mean ± SEM (*N* = 6)

Histological analysis of the frozen-thawed testicular tissue revealed considerable tissue damage compared to fresh tissue (Fig. 4C, D). The testicular tissue sections from CFM as well as LFM exhibited detachment of testicular cells from the basement membrane (*p* < 0.01) and poor tubular integrity compared to the fresh tissue. However, vimentin-positive cells per tubule were observed to be significantly low (*p* < 0.0001) in tissue cryopreserved with CFM compared to the fresh tissue. However, freezing in LFM seems to retain a comparable percentage of vimentin-positive cells (Fig. 4E, F). Further, when we tested the beneficial properties of LFM in cryopreservation of adult human testicular tissue, similar results were obtained. Cryopreservation in LFM non-significantly increased cryosurvival, prevented DNA damage (*p* < 0.01), and decreased apoptosis (*p* < 0.01) in comparison to tissues frozen in CFM (Fig. 5A-E).

### Lipid Uptake by Testicular Tissue from Liposome During Prepubertal Testicular Tissue Cryopreservation

To understand if the liposomes interact with the testicular cells during the freeze–thaw process, testicular tissue was incubated with liposomes encapsulated FITC (Fig. 5F). Microscopic imaging after thawing the tissue revealed that 48.52 ± 5.6% of testicular cells had taken up FITC (Fig. 5G, H), suggesting that the liposomes interact with testicular cells during the freeze–thaw process.

### Effect of Various Penetrating Cryoprotectants Individually or in Combinations, on Cryopreservation of Prepubertal Testicular Tissue Using LFM by Slow Freezing Method

The prepubertal testicular tissue cryopreserved in a liposome-based medium containing 5% DMSO exhibited 72.73 ± 1.42% viable cells and 23.15 ± 2.11% cells with DNA damage. Among the penetrating cryoprotectants, DMSO (5%) was found to offer maximum cryoprotective effect, when used alone compared to glycerol (10%), propanediol (10%), and ethylene glycol (10%, Table 3). A significant decrease in viability was observed when 5% DMSO was replaced with either glycerol (*p* < 0.0001), propanediol (*p* < 0.0001), or ethylene glycol (*p* < 0.01). Similarly, an increase in DNA damage was observed in the tissue cryopreserved in glycerol (*p* < 0.05), propanediol (*p* < 0.0001), and ethylene glycol (non-significant) alone compared to the tissue frozen in medium containing 5% DMSO. The order of efficiency of penetrating cryoprotectant was DMSO > ethylene glycol > glycerol > propanediol.

**Table 3:** Effect of various concentrations of DMSO, Glycerol, propanediol (PD), and ethylene glycol (EG) alone or in combination on the viability and DNA damage of testicular cells cryopreserved with liposome-based freezing medium (*N* = 6)

Groups	Details	Penetrating cryoprotectant used (%)				Viability (%)	DNA damage (%)
		DMSO	Glycerol	PD	EG		
Fresh tissue		-	-	-	-	87.92 ± 0.79	10.87 ± 2.02
Cryopreserved in freezing medium with single penetrating cryoprotectant	DMSO	5.0	-	-	-	72.73 ± 1.42	23.15 ± 2.11**
	Glycerol	-	10.0	-	-	44.28 ± 0.98****	33.70 ± 1.12*
	Propanediol	-	-	10.0	-	41.35 ± 3.11****	49.55 ± 2.70****
	Ethylene glycol	-	-	-	10.0	54.91 ± 6.27**	33.66 ± 0.69
Cryopreserved in freezing medium with combination of penetrating cryoprotectants	DMSO + Glycerol	4.0	1.0	-	-	71.55 ± 1.10	26.55 ± 1.51
		2.5	2.5	-	-	65.42 ± 2.75	28.52 ± 1.85
		5.0	2.5	-	-	64.75 ± 1.74*	26.37 ± 1.04
		2.5	5.0	-	-	61.70 ± 0.96***	31.68 ± 1.96
		2.5	7.5	-	-	60.40 ± 1.72**	47.18 ± 4.16****
		5.0	10.0	-	-	29.72 ± 2.14****	49.85 ± 1.96****
	DMSO + Propanediol	4.0	-	1.0	-	67.41 ± 2.17	34.20 ± 2.15
		2.5	-	2.5	-	57.78 ± 2.53**	41.90 ± 1.42
		5.0	-	2.5	-	60.96 ± 1.93	48.23 ± 2.91***
		2.5	-	5.0	-	42.55 ± 1.38****	54.18 ± 0.57****
		2.5	-	7.5	-	43.15 ± 3.29****	50.25 ± 3.24****
		5.0		10.0	-	45.93 ± 1.96****	40.78 ± 2.12
	DMSO + Ethylene Glycol	4.0	-	-	1.0	63.96 ± 3.16	25.25 ± 1.76
		2.5	-	-	2.5	64.43 ± 1.33	32.91 ± 1.85
		5.0	-	-	2.5	67.06 ± 1.63	26.31 ± 1.16
		2.5	-	-	7.5	60.10 ± 2.31	27.05 ± 0.46
		2.5	-	-	5.0	64.05 ± 0.85	30.71 ± 5.45
		5.0	-	-	10.0	61.11 ± 1.81	34.41 ± 1.13

The data represents Mean ± SEM. *p < 0.05, **p < 0.01, ***p < 0.001, ****p < 0.0001 v/s DMSO

When we assessed the outcome of cryopreservation in tissues frozen with various combinations of two different types of penetrating cryoprotectants, the best results were observed in prepubertal testicular tissue frozen in a medium containing 1% glycerol and 4% DMSO, which was comparable to 5% DMSO alone group. Further increase in the concentration of glycerol (2.5 to 10%) resulted in a dose-dependent increase in cell death and DNA damage in testicular cells compared to 5% DMSO alone. Similar results were observed when prepubertal testicular tissue was frozen in a freezing medium with propanediol and DMSO. Cryopreservation with 1% propanediol and 4% DMSO showed comparable results to tissue cryopreserved with 5% DMSO. Higher concentrations of propanediol (7.5–10%) with or without DMSO (2.5–5%) resulted in significant (*p* < 0.0001) cell toxicity. Compared to different concentrations of glycerol and propanediol in combination with DMSO, cryopreservation with various concentrations of ethylene glycol resulted in higher cell viability across all the combinations tested. Further, DNA damage was lower in the various combinations of ethylene glycol tested compared to glycerol and propanediol. However, the combination of different concentrations of ethylene glycol and DMSO did not improve the freeze–thaw outcome during prepubertal testicular tissue cryopreservation compared to the 5% DMSO alone group (Table 3).

## Discussion

In this study, we report the successful formulation of a liposome-based freezing medium optimized for cryopreservation of prepubertal testicular tissue using a slow freezing protocol. The formulation works effectively for prepubertal mice and demonstrates a significant beneficial effect of this formulation over the conventional DMSO-based freezing medium. Further, our findings confirm that among the commonly used penetrating cryoprotectants, 5% DMSO was observed to be optimum for prepubertal testicular tissue cryopreservation.

Freeze–thaw process of prepubertal testicular tissue is known to result in loss of cell viability [33, 34]. In the current study, exogenous supplementation of 0.25 mg/mL of liposomes improved the post-thaw survival of prepubertal testicular cells, indicating that liposomes have a protective role during cryopreservation. An earlier study from our group demonstrated that liposomes made up of soy lecithin and cholesterol improved post-thaw human sperm parameters compared to the conventional chicken egg yolk-based medium [20]. Supplementation of lipid extract [35], or liposomes with [18, 19] or without [20, 36] antioxidants have been observed to be highly beneficial during cryopreservation. In literature, liposomes have been used for cryopreserving sperm of different species [20, 37, 38], coral larvae [39], human hematopoietic cell lines [21], and red blood cells [40]. However, to the best of our knowledge cryopreservation of prepubertal mice testicular tissue using liposomes has not been reported so far.

The plasma membrane is the prime target for cryoinjury when cells are exposed to low temperatures [41]. In addition to impairing cellular macromolecular function, the oxidative stress generated is reported to result in the loss of membrane phospholipids and cholesterol [6–8], rearrangement of lipid species [9, 10], and altering membrane protein function [5]. Therefore, exogenous supplementation of lipids in the form of liposomes can be a potent approach for increasing cell functioning post-freeze–thaw process and protecting them from cryoinjury. In the testicular cells, the oxidative stress-induced damage is combated by the GSH, superoxide dismutase (SOD), and the glutathione peroxidase/reductase system (GPX/GRD). In the current study, the significant increase in MDA and decrease in reduced GSH levels, and the increase in mRNA transcripts of *Sod1*, *Cat*, and *Gpx4* in tissue cryopreserved in CFM confirms that the testicular tissue is under oxidative stress, which is mitigated by the addition of liposomes. In addition to these changes, excess reactive oxygen species (ROS), and reactive nitrogen species (RNS), generated during the freeze–thaw process activate mitochondrial proteins like BCL-2, which triggers the release of proapoptotic substances from the mitochondria into the cytoplasm, leading to apoptosis [24]. Abdelnour et al. [18] observed that supplementation of propolis-loaded nanoliposomes decreased *Bax* and *Caspase-3* mRNA levels in buffalo sperm after the freeze–thaw process. Further, Mohammadzadeh et al. [42] reported improved sperm parameters and reduced apoptosis in human spermatozoa post-thaw when nanoliposomes were supplemented into the freezing medium. The significantly lower percentage of Annexin V-positive testicular cells and, significantly higher expression of *Bcl-2*, lower expression of *Bax*, *Cyt c,* and *Caspase-3* mRNA in the tissue frozen with LFM suggest that the supplementation of liposomes in the freezing medium effectively prevented freeze–thaw-induced apoptosis. However, in the current study liposomes loaded with antioxidants did not further reduce the cryosusceptibility of prepubertal testicular tissue to freeze–thaw-induced damage compared to the liposome alone group (Fig S1B, D, Supplementary information).

Preserving testicular tissue integrity after the freeze–thaw process is crucial for maintaining the intricate crosstalk between the somatic (Leydig and Sertoli) and germ cells [43] for spermatogenesis to commence after transplantation or in vitro tissue culture [44]. Damaged basement membrane, reduction in the number of intact seminiferous tubules, interstitial fibrosis, and a decrease in intratubular cell density [43, 45] are consequences of the freeze–thaw process on testicular architecture. Although the intertubular distance and basal membrane damage were unaltered compared to the control, the higher expression of vimentin in testicular tissue cryopreserved in LFM implies that the presence of liposomes in the freezing media helps to preserve tissue architecture.

It is crucial to note that the mechanisms underlying the interaction between cell membranes and liposomes are intricate and vary depending on the composition and characteristics of the liposomes. In the current study, the uptake of FITC by the testicular cells after the freeze–thaw process confirms that the liposomes help in testicular cell membrane rebuilding. Hence, the protective effect of the liposome could be due to changes in the phospholipid-to-protein and cholesterol ratios or the ratio of polyunsaturated to saturated lipids in the membrane due to the interaction of the liposomes with the testicular cells. It is also plausible as demonstrated in other cells, that reversible binding of phospholipid structures to the surface of the cell membrane, rearranges the components of the membrane [46]. As demonstrated by Arts et al. [47], the fusion of spermatozoa with liposomes requires negatively charged phospholipids, whereas the fusion reaction is inhibited when phosphatidylcholine, a neutral lipid, is incorporated into the vesicle. Furthermore, these authors demonstrated that the condition of the spermatozoa affected fusion, as fresh cells showed poor response to facilitate liposome fusion, whereas liposomes readily fused with frozen-thawed cells. In the current study, the mitigation of freeze–thaw-induced damage of testicular tissue in the liposome-supplemented group could be due to the composition of the liposomes. The presence of neutral phosphatidylcholine may stabilize the membrane, while phosphatidylethanolamine at low concentrations may enable cell-liposome fusion [48]. Earlier studies have reported the beneficial effect of the presence of liposomes made of phosphatidylserine alone or in combination with cholesterol on the fertilization outcome of stallion sperm post-cryopreservation [49, 50]. Further, cholesterol is typically utilized as the main steroid in liposome synthesis at a ratio of less than 30% of total lipids to enhance the rigidity and stability of the liposomes [51]. Indeed, cholesterol modulates liposome size, morphology, and fluidity [52], which is advantageous for cryopreservation. In the current study, liposomes prepared with phosphatidylethanolamine, and optimum phospholipids to cholesterol ratio might have an added advantage on the outcome of the prepubertal testicular tissue cryopreservation. This composition further explains the interaction of the formulated liposomes and the uptake of fluorescent dye by the testicular cells during the freeze–thaw process. Further, the lower freezing point of LFM compared to CFM, may provide the advantage to the tissues by providing additional time to undergo dehydration, enhancing the cryopreservation outcome.

The choice of penetrating cryoprotectant in the freezing medium is critical in minimizing the intracellular ice crystal formation and mitigating the oxidative stress generated during the freeze–thaw process. The effectiveness of the penetrating cryoprotectant in improving cryosurvival depends upon the diffusion coefficient of the penetrating cryoprotectant [53]. Therefore, the type of penetrating cryoprotectant used in the freezing medium differs for different cell types subjected to cryopreservation [54]. Since the prepubertal testicular tissue has a heterogeneous cell population comprised of germ cells and somatic cells, it is challenging to formulate a freezing medium with penetrating cryoprotectant(s) suitable for both these cell types. Beckman and Coniglio [55] have demonstrated that the lipid composition is different for somatic and germ cells of testicular tissue, possibly resulting in differential response to cryopreservation-induced challenge.

Using the murine model, Goossens et al. [56] reported that the structure of the seminiferous tubules in prepubertal testicular tissue is better preserved when cryopreserved in a medium containing DMSO compared to ethylene glycol. Further, a report by Unni et al. [54] suggests that DMSO is effective for cryopreservation of immature testicular tissue, whereas ethylene glycol is effective for adult testicular tissue. These results suggest that DMSO is a suitable penetrating cryoprotectant for prepubertal testicular tissue cryopreservation. Further, from the previous reports it is evident that DMSO is the most suitable penetrating cryoprotectant for testicular tissue cryopreservation [1] when used in the range of 5–10% [43, 54]. Gouk et al. [33] reported that cryopreservation of immature mouse testicular tissue with DMSO is superior and has lower cytotoxicity than glycerol, propanediol, and ethylene glycol. Our findings agree with previous literature, which reports the superior effect of DMSO in protecting the testicular tissue when compared to ethylene glycol [54, 57], propanediol [26, 58], and glycerol [57, 58]. The use of a combination of penetrating cryoprotectants has been attempted earlier to improve the cryosurvival of cells and tissues [59, 60]. However, formulation of tissue-specific freezing medium containing a combination of penetrating cryoprotectants is challenging as the selection of penetrating cryoprotectants should be based on the diffusion coefficient and lower cytotoxicity [53]. The freezing medium comprising of combination of penetrating cryoprotectants in which the total concentration exceeded 5% was detrimental to testicular tissues. Similar observations have been reported earlier [54, 61].

In the current study, apart from DMSO, ethylene glycol alone or in various combinations with DMSO did not affect viability and DNA damage significantly. These results agreed with a previous study by Silva et al. [59], where cryopreservation of Adult Red-Rumped Agoutis testicular tissue with DMSO or ethylene glycol alone or in combination did not yield any difference in viability. Further, the inappropriate choice of penetrating cryoprotectant could result in changes in the physiological properties of the liposomes causing instability. Cryoprotective agent-liposome interactions have been studied earlier in detail using both experimental and molecular dynamic simulations [62] and have found DMSO to be suitable for use in a freezing medium containing liposomes. The highest cryoprotective effect observed for DMSO-based freezing medium in the present study further confirms that the presence of DMSO in the freezing medium confers stability to liposomes in addition to its cryoprotective role.

## Conclusion

The current formulation offers impressive cryosurvival for mice prepubertal testicular cells. This formulation can be tested for the beneficial outcome on the cryopreservation of other essential organs, tissues, or cells considering the mechanism of action of the established freezing medium. One major limitation of this work is that we have not examined the potential benefits of liposomes on the testicular tissue cryopreservation of prepubertal boys. Furthermore, the proliferation and differentiation ability of the germ cells obtained from tissue cryopreserved in LFM has not been studied. However, the present investigation has provided sufficient experimental evidence in support of the beneficial properties of liposomes of membrane lipid components in enhancing the prepubertal testicular cells.

## Supplementary Information

Below is the link to the electronic supplementary material.

Supplementary file1 (DOCX 268 KB)

## Data Availability

All data generated or analyzed during this study are included in this published article and its Supplementary Information files.
