# Supplementary material for: Liposome-based Freezing Medium Improves the Outcome of Mouse Prepubertal Testicular Tissue Cryopreservation
Source: Reprod Sci. 2024 Sep 19;31(11):3532–48. doi: 10.1007/s43032-024-01688-4 (PMC11527951; doi:10.1007/s43032-024-01688-4)
Supplement: Supplementary file 1 — Supplementary file1 (DOCX 268 KB) [file 43032_2024_1688_MOESM1_ESM.docx]

**Liposome-based Freezing Medium Improves the Outcome of Mouse Prepubertal Testicular Tissue Cryopreservation**

Reyon Dcunha^1^, Sadhana P Mutalik^2^, Reethu Ann Reji^3^, Srinivas Mutalik^2^, Sneha Guruprasad Kalthur^4^, Padmaraj Hegde^5^, M S Murari^6^, Shamprasad Varija Raghu^7^, Shreetama Banerjee^8^, Anujith Kumar^8^, Satish Kumar Adiga^3^, Yulian Zhao^9, 10^, Nagarajan Kannan^10,11,12^, Guruprasad Kalthur^13,#^

^1^Division of Reproductive Genetics, Department of Reproductive Science, Kasturba Medical College, Manipal, Manipal Academy of Higher Education, Manipal- 576104, Karnataka, India

^2^Department of Pharmaceutics, Manipal College of Pharmaceutical Sciences, Manipal Academy of Higher Education, Manipal- 576104, Karnataka, India

^3^Centre of Excellence in Clinical Embryology, Department of Reproductive Science, Kasturba Medical College, Manipal, Manipal Academy of Higher Education, Manipal- 576104, Karnataka, India

^4^Department of Anatomy, Kasturba Medical College, Manipal, Manipal Academy of Higher Education, Manipal- 576104, Karnataka, India

^5^Department of Urology, Kasturba Medical College, Manipal, Manipal Academy of Higher Education, Manipal, Manipal- 576104, Karnataka, India.

^6^DST PURSE Program, Mangalore University, Mangalagangotri, Mangalore, 574199, Karanatka, India

^7^Division of Neuroscience, Yenepoya Research Centre (YRC), Yenepoya (Deemed to be University), Mangalore- 575018, Karnataka, India.

^8^Manipal Institute of Regenerative Medicine, Bangalore, Manipal Academy of Higher Education, Manipal- 560064, Karnataka, India.

^9^Division of Reproductive Endocrinology and Infertility, Department of Obstetrics and Gynecology and Department of Laboratory Medicine and Pathology, Mayo Clinic, Rochester- 55902, Minnesota, USA.

^10^Department of Laboratory Medicine and Pathology, Mayo Clinic, Rochester- 55905, Minnesota, USA

^11^Center for Regenerative Biotherapeutics, Mayo Clinic, Rochester- 55905, Minnesota, USA

^12^Mayo Clinic Comprehensive Cancer Center, Mayo Clinic, Rochester- 55905, Minnesota, USA

^13^Division of Reproductive Biology, Department of Reproductive Science, Kasturba Medical College, Manipal, Manipal Academy of Higher Education, Manipal- 576104, Karnataka, India

**^#^Corresponding author:**

Dr Guruprasad Kalthur, MSc, PhD

Head, Division of Reproductive Biology

Head, Department of Reproductive Science

Kasturba Medical College, Manipal,

Manipal Academy of Higher Education, Manipal 576104, India

Electronic Mail: [guru.kalthur@manipal.edu](mailto:guru.kalthur@manipal.edu)

ORCID ID: 0000-0002-4554-2917

**Table S1:** List of primers and probes used for qRT-PCR.

| **Gene** | **Primers** | |
| --- | --- | --- |
|  | **Forward primer** | **Reverse primer** |
| *Gapdh* | AGGTCGGTGTGAACGGATTTC | TGTAGACCATGTAGTTGAGGTCA |
| *P53* | GACCGCCGTACAGAAGAAGA | GCGGATCTTGAGGGTGAAATA |
| *Bax* | ATCTGGTTCTGCAAGCGTTTA | CCTGCTCCGAATTTGGTGAAA |
| *Bcl-2* | ATGCCTTTGTGGAACTATATGGC | GGTATGCACCCAGAGTGATGC |
| *Cyt C* | CAGCTTCCATTGCGGACAC | GGCACTCACGGCAGAATGAA |
| *Caspase-3* | ATGGAGAACAACAAAACCTCAGT | TTGCTCCCATGTATGGTCTTTAC |
| **Gene** | **Assay ID of TaqMan® assay probes.** | |
| *Gapdh* | Mm99999915_g1 | |
| *Gpx4* | Mm00515041_m1 | |
| *Catalase* | Mm00437992_m1 | |
| *Sod1* | Mm01344233_g1 | |

**Elucidation of optimum liposome concentration in the freezing medium**

For optimization of liposomes for prepubertal testicular tissue, the testicular tissue was collected from prepubertal mice in DMEM/F12 (11320033, Gibco, USA) medium on ice. Immediately after collection, the testis was decapsulated and cut into 3 mm^2^ pieces. The testicular tissue was then transferred to a cryovial (P60116, Abdos, USA) containing 500 μL of either control freezing medium (CFM) composed of 5% dimethyl sulfoxide (DMSO, D4540, Sigma, USA) and 30% fetal bovine serum (FBS, CCS-500-SA-U, Genetix Biotech, Cell clone^TM^, India) in DMEM/F12 medium or different concentrations of simple (0.05, 0.1, 0.25, 0.5, 0.75, 2.5, 5.0 mg/ mL) or complex (0.05, 0.1, 0.25, 0.5, 0.75, 2.5 mg/ mL) liposomes. The cryovials were then placed in an isopropanol chamber (Mr. Frosty^TM^ Freezing Container, 5100-0001, Thermofisher Scientific, USA) at -80ºC for 24 h and then stored in liquid nitrogen (LN_2_) for a minimum of one week.

Thawing of testicular tissue was performed according to the protocol described by Milazzo et al [1], with minor modifications. Briefly, the samples were thawed rapidly by placing the cryovials in a water bath maintained at 37ºC for 2 min. Tissues were then placed sequentially in thawing solution 1 (TS1; 2.5% DMSO, 0.05 M sucrose and 10% FBS in DMEM/F12 medium) followed by thawing solution 2 (TS2; 1% DMSO, 0.05 M sucrose and 10% FBS in DMEM/F12 medium), thawing solution 3 (TS3; 0.05 M sucrose in DMEM/F12 medium) and finally in thawing solution 4 (TS4; DMEM/F12 Medium) for 5 min each at room temperature. The tissues were kept on ice until further handling.

Simple and complex liposomes were assessed for their cryoprotective effect on prepubertal testicular tissue by assessing cell viability and DNA quality (Fig. S1A-D). A significant decrease in cell viability (P<0.001) was observed in the testicular tissues cryopreserved in CFM, compared to the fresh tissue. However, supplementation of 0.25 mg/mL of simple and complex liposomes demonstrated a significant increase (p<0.05) in viability compared to CFM (Fig. S1A, B). Although the presence of 0.1 mg/mL of simple liposomes resulted in a non-significant increase in viability, the addition of 0.1 mg/mL complex liposome to the freezing medium demonstrated a significant (p<0.05) better survival. Further, cryopreserving testicular tissues in CFM resulted in a significant increase (p<0.0001) in the percentage of γ-H2AX-positive cells compared to unfrozen tissue. For both simple and complex liposomes, the optimum cryoprotective effect of LFM was observed at 0.1 and 0.25 mg/mL concentrations (p<0.0001 for simple liposomes; p<0.01 at 0.1 mg/mL and p<0.001 at 0.25 mg/mL vs complex liposomes, Fig. S1C, D). However, there was no considerable difference in the viability and γ-H2AX expression between 0.25 mg/mL of complex liposomes and tissues cryopreserved with simple liposomes. Hence, 0.25 mg/mL of simple liposomes was considered as optimum concentration and used for further experiments (LFM).

BA

A

DA

CA

**Figure S1:** Optimization of liposomes for prepubertal testicular tissue cryopreservation. **A)** Effect of different concentrations of simple liposomes on the viability of prepubertal testicular cells post-freeze-thaw process (N=6); ***p<0.001 v/s Fresh, *p<0.05 v/s CFM. **B)** Effect of different concentrations of complex liposomes on the viability of testicular cells post-freeze-thaw process (N=6); ****p<0.0001 v/s Fresh, *p<0.05 v/s CFM **C)** Effect of different concentrations of simple liposomes on the expression of γ-H2AX in prepubertal testicular cells post-freeze-thaw process; ****p<0.0001 v/s Fresh, ***p<0.001, ****p<0.0001 v/s CFM. **D)** Effect of different concentrations of complex liposomes on the expression of γ-H2AX in prepubertal testicular cells post-freeze-thaw process. ****p<0.0001 v/s Fresh, **p<0.01, ***p<0.001 v/s CFM. The data is represented as Mean ± SEM.

**Reference**

[1] J.P. Milazzo, L. Vaudreuil, B. Cauliez, E. Gruel, L. Massé, N. Mousset-Siméon, B. Macé, N. Rives, Comparison of conditions for cryopreservation of testicular tissue from immature mice, Human Reproduction 23 (2008) 17–28. https://doi.org/10.1093/humrep/dem355.
